# Health financing policy responses to the COVID-19 pandemic: a review of the first stages in the WHO South-East Asia Region

**DOI:** 10.1093/heapol/czac071

**Published:** 2022-09-06

**Authors:** Thomas Gadsden, Belinda Ford, Blake Angell, Bojan Sumarac, Valeria de Oliveira Cruz, Hui Wang, Tsolmon Tsilaajav, Stephen Jan

**Affiliations:** The George Institute for Global Health, University of New South Wales, Level 5/1 King Street, Newtown, Sydney, New South Wales 2042, Australia; The George Institute for Global Health, University of New South Wales, Level 5/1 King Street, Newtown, Sydney, New South Wales 2042, Australia; The George Institute for Global Health, University of New South Wales, Level 5/1 King Street, Newtown, Sydney, New South Wales 2042, Australia; The George Institute for Global Health, University of New South Wales, Level 5/1 King Street, Newtown, Sydney, New South Wales 2042, Australia; Health Financing and Governance, World Health Organization South-East Asia Regional Office, World Health House, Indraprastha Estate, Mahatma Gandhi Marg, New Delhi 110 002, India; Health Financing and Governance, World Health Organization South-East Asia Regional Office, World Health House, Indraprastha Estate, Mahatma Gandhi Marg, New Delhi 110 002, India; Health Financing and Governance, World Health Organization South-East Asia Regional Office, World Health House, Indraprastha Estate, Mahatma Gandhi Marg, New Delhi 110 002, India; The George Institute for Global Health, University of New South Wales, Level 5/1 King Street, Newtown, Sydney, New South Wales 2042, Australia; Health Financing and Governance, Faculty of Medicine and Health, The University of Sydney School of Public Health, Sydney, New South Wales 2006, Australia

**Keywords:** COVID-19, health system financing, financial protection, South-East Asia

## Abstract

COVID-19 imposed unprecedented financing requirements on countries to rapidly implement effective prevention and control measures while dealing with severe economic contraction. The challenges were particularly acute for the 11 countries in the WHO South-East Asia Region (SEAR), home to the lowest average level of public expenditure on health of all WHO regions. We conducted a narrative review of peer-reviewed, grey literature and publicly available sources to analyse the immediate health financing policies adopted by countries in the WHO SEAR in response to COVID-19 in the first 12 months of the pandemic, i.e. from 1 March 2020 to 1 March 2021. Our review focused on the readiness of health systems to address the financial challenges of COVID-19 in terms of revenue generation, financial protection and strategic purchasing including public financial management issues. Twenty peer-reviewed articles were included, and web searches identified media articles (*n* = 21), policy reports (*n* = 18) and blog entries (*n* = 5) from reputable sources. We found that countries in the SEAR demonstrated great flexibility in responding to the COVID-19 pandemic, including exploring various options for revenue raising, removing financial barriers to care and rapidly adapting purchasing arrangements. At the same time, the pandemic exposed pre-existing health financing policy weaknesses such as underinvestment, inadequate regulatory capacity of the private health sector and passive purchasing, which should give countries an impetus for reform towards more resilient health systems. Further monitoring and evaluation are needed to assess the long-term implications of policy responses on issues such as government capacity for debt servicing and fiscal space for health and how they protect progress towards the objectives of universal health coverage.

Key messages

- This review provides a snapshot of immediate health financing policy responses to the COVID-19 pandemic in the WHO South-East Asia Region from 1 March 2020 to 1 March 2021.
- Countries demonstrated great flexibility and agility in responding to the COVID-19 pandemic, including exploring various options for revenue raising, removing financial barriers to care and adapting purchasing arrangements.
- Health systems that had well-developed institutions for strategic purchasing (including engaging with the private sector) were shown to be able to respond more rapidly and effectively in harnessing this capacity to support the COVID-19 response.
- Nevertheless, the sheer number of emergency announcements to expand the funding and capacity of the health system demonstrate that the pandemic highlighted pre-existing health financing policy weaknesses such as underinvestment and passive purchasing, which should give countries an impetus for reform.

## Introduction

From March 2020, the COVID-19 pandemic triggered a global health and economic crisis. Countries faced unprecedented financing requirements to rapidly implement prevention and control measures and mitigate the immediate financial pressures faced by households and businesses through extraordinary spending programmes. At the same time, the global economy slowed, sharply reducing government revenues and spending capacity. As of 1 March 2021, COVID-19 has cost more than 2.5 million lives (Kurowski *et al.*, 2021).

The challenges were particularly acute for the 11 countries in the WHO South-East Asia Region (SEAR), which is home to over a quarter of the world’s population including some of the largest countries in the world such as India, Indonesia and Bangladesh, and some of the smallest in Bhutan, Maldives and Timor-Leste (World Health Organization, 2020a). Other countries in the region include the Democratic People’s Republic of Korea, Myanmar, Nepal, Sri Lanka and Thailand. Compared to other WHO regions, the SEAR has the lowest average level of public expenditure on health yet, prior to the pandemic, was one of the fastest growing with an average Gross Domestic Product (GDP) growth rate of 4.1% per capita annually from 2009 to 2019 (World Health Organization, 2019; World Bank, 2020). The SEAR has also made vast gains in population health and health service coverage over the past decade yet still had one of the highest poverty rates in the world (World Bank, 2020).

The impact of the COVID-19 pandemic in the SEAR has been severe—the region had experienced approximately one-fifth of global COVID-19 cases as of 11 June 2021 (World Health Organization, 2021). An estimated 60% of essential services were at least partially disrupted in 2020 due to social distancing and lockdown policies (World Health Organization, 2020c). It is estimated that COVID-19 will push an additional 48–59 million people in the SEAR into extreme poverty in 2020 (Lakner *et al.*, 2021). These challenges have placed further strain on already stretched health spending in the region. Alongside health financing system indicators, Table 1 presents the cumulative number of confirmed cases and deaths in each SEAR country on 1 March 2021.

**Table 1.** Health financing system indicators and cumulative number of COVID-19 cases and deaths by country by 1 March 2021

Country	Out-of-pocket expenditure as % of current health expenditure (2019)	General government health expenditure as % of current health expenditure (2019)	General government health expenditure as % of GDP (2019)	Number of COVID-19 cases (million)	Number of COVID-19 deaths
Bangladesh	72.7	18.6	0.5	546 801	8423
Bhutan	17.8	73.6	2.7	867	1
India	54.8	32.8	1.0	11.12	157 346
Indonesia	34.8	48.9	1.4	1.34	36 518
Maldives	16.5	79.3	6.4	19 979	62
Myanmar	76.0	15.8	0.7	141 916	3199
Nepal	57.9	24.8	1.1	274 216	2777
Sri Lanka	45.6	47.2	1.9	83 552	483
Thailand	8.7	71.7	2.7	26 144	84
Timor-Leste	8.2	55.9	4.0	113	0

Source: Dong *et al.* (2020), World Health Organization.

There is much to be learnt from reviewing the readiness of health systems and ability to enact mitigation measures to respond to these shocks. Health financing policy will need to focus on ensuring affordable access to health services for poor and vulnerable populations, while also putting in place critical systems to manage COVID-19 (Sparkes *et al.*, 2021). Recommendations to achieve this include (1) delinking health insurance benefits from employment, (2) prioritizing services for vulnerable population groups including people living below the poverty line and (3) prioritizing common goods for health such as cross-border surveillance (Yazbeck *et al.*, 2020; Kurowski *et al.*, 2021; Sparkes *et al.*, 2021).

This review provides a snapshot of the immediate health financing policy responses to the COVID-19 pandemic by countries in the WHO SEAR, in the first 12 months from 1 March 2020 to 1 March 2021. This study was undertaken in the context of rapid policy change with the purpose of informing response efforts by WHO. Reviewing the immediate response to such a crisis provides an important perspective on the ability of each country to mobilize and translate resources into effective services and treatments on the ground, while maintaining affordable access for poor and vulnerable populations. It is also critical to shed light on the policies and existing institutional factors that enable effective emergency responses and ultimately safeguard progress towards universal health coverage in the coming years.

## Methods

### Approach for the study

This study was commissioned by WHO to identify health financing policies announced or implemented by countries in the WHO SEAR in response to the COVID-19 pandemic from March 2020 to March 2021. A scoping review with narrative synthesis was deemed the most appropriate methodology to capture information from a variety of sources (Green *et al.*, 2006; Ferrari, 2015). Similar methods have been used elsewhere to understand experiences of emerging outbreaks (Ogoina, 2015). Our review focused on policy responses to the pandemic that fell under the health financing functions and goals of revenue generation, financial protection and strategic purchasing including public financial management issues. Specific evaluation questions of interest are shown in Table 2. Vaccine financing among SEAR countries was assessed through a complementary review (Yap, 2021).

**Table 2.** Outcomes of interest by health financing function/goal

Health financing function/goal	Outcomes of interest
Revenue generation	How much funding did the country allocate (and/or reallocate) to the health sector specifically for coping with COVID-19?
Financial protection	Did the country allow for out-of-pocket fees to be charged for COVID-related tests and treatments? Was there a temporary suspension in user fees for other non-COVID-related services during the time of the pandemic? Were any new financial protection schemes created to cover COVID-related services to groups that would otherwise risk financial hardship?
Purchasing mechanisms	How were providers paid? Were new accreditation procedures established for facilities to provide care to COVID-19 patients? Were actions taken to accelerate fund release to frontline service providers?

### Search strategy

A systematic search of the PubMed database was conducted using MeSH headings and permutations of the search terms contained in Box 1 and was limited to English language peer-reviewed articles published from March 2020 to March 2021. Grey literature was searched using multiple complementary strategies to minimize the risk of omitting relevant sources. This included using the search terms provided in Box 1 in combination with each country name, or applying country filters, and limited to years 2020–21 to search the following targeted websites: the WHO, World Bank (filtered for Southeast Asia), Asian Development Bank, International Monetary Fund (IMF) and Centre for Global Development. Where available, Ministries of Finance and Health websites for each country were searched using the same strategy. Next, multiple combinations of search terms from Box 1 (e.g. ‘covid’ AND health financing AND ‘Thailand’) and limited to years 2020–21 were used to search Google for media reports and additional open-source documentation. Search ‘hits’ that were identified as potentially relevant were retained for further screening. Lastly, consultation with key informants from WHO country offices in the SEAR confirmed relevance and appropriateness of our sources.

**Box 1.** PubMed search terms

1. “COVID-19” OR “COVID” OR “Coronavirus” OR “SARS-CoV-2” 2. “Bangladesh” OR “Bhutan” OR “India” OR “Indonesia” OR “Maldives” OR “Myanmar” OR “Nepal” OR “Sri Lanka” OR “Thailand” OR “Timor Leste” 3. “Cost*” OR “Fee*” OR “financ*” OR “Fund*” OR “Revenue*” OR “Pay*” OR “Benefit*” 4. “Health” OR “health financ*” OR “policy” OR “benefit package” OR “financial protection” OR “public financial management” 5. 3 or 4 6. 1 and 2 and 5 Additional filters applied include: Only studies related to humans Only studies published since 1 January 2020

A snowball search method was also used to track down details of financing policies specific to each country. For example, if a particular policy response was identified in early searches, the name of the programme would be used as a later search term. As information was changing on a daily basis during the initial phases of the pandemic, this method was particularly important to uncover media reports that clarified government policy announcements, prior to formal peer review evaluation.

### Eligibility criteria

Eligibility was limited to sources that described health financing policies announced or implemented by countries in the WHO SEAR in response to the COVID-19 pandemic from 1 March 2020 to 1 March 2021. Peer-reviewed articles were included if the title and/or abstract indicated the report of information relevant to the outcomes of interest in Table 2. Studies in non-SEAR settings or studies that reported only qualitative results were excluded, as were opinions and letters.

Media reports and blogs were only included following appraisal of the website and content by the reviewers, which considered the credibility of the source and authors, such as local news outlets or blogs by health policy experts. These reports often used language targeted at the general population and thus were cross-referenced for accuracy against other formal sources, such as peer-reviewed articles or government policy briefings. Additional advice was sought from WHO country experts to ensure only quality evidence was included.

### Screening

All peer-reviewed articles identified in the search were imported into Endnote, which was used for title and abstract screening. Duplicates were removed from the initial search. Abstracts and potentially relevant full texts were reviewed independently by three authors with any conflicts resolved by consensus. Thereafter, full texts of potentially relevant studies were reviewed to determine eligibility for inclusion.

### Data extraction and synthesis

Data were extracted by three authors into a spreadsheet with key variables including country, date of policy announcement, health financing function and outcome of interest. Due to the heterogeneity of the methods of included studies, no formal quality assessment was carried out of reviewed studies. A narrative synthesis was conducted to describe and interpret findings in relation to the outcomes of interest.

## Results

In total, 20 studies were included in this review (Anwar *et al.*, 2020; Piryani *et al.*, 2020; Upadhaya *et al.*, 2020; Thiagarajan, 2020; Cousins, 2020; Al-Zaman, 2020; Olivia *et al.*, 2020; de Jong and Ho, 2020; Ayuningtyas *et al.*, 2021; Gauttam *et al.*, 2021; Jose *et al.*, 2020; Joseph *et al.*, 2021; Kabaniha *et al.*, 2021, Kumar and Pinky; Rao *et al.*, 2021; Safitri *et al.*, 2021; Williams *et al.*, 2021; Roziqin *et al.*, 2021; Sparrow *et al.*, 2020). Excluded studies included epidemiological accounts of localized outbreaks, case studies and accounts from clinicians. Web searches and snowballing identified a larger number of relevant sources, including grey literature (*n* = 18), government reports (*n* = 4), news media (*n* = 21) and blog entries (*n* = 5) from reputable sources (i.e. World Bank, Centre for Global Development, P4H Network and IMF).

Most peer-reviewed and grey literature was found for India (*n* = 14), followed by Bangladesh (*n* = 10), Indonesia (*n* = 9) and Nepal (*n* = 9). Limited information was available for Maldives, Bhutan, Myanmar and Timor-Leste and none was found on the Democratic People’s Republic of Korea. The key findings of this search are summarized below under each of the three key domains.

### Domain 1: resource generation for the health sector

In the first year of the pandemic, all SEAR countries announced exceptional budget allocations to stimulate the economy and rapidly expand the capacity of the health system. Common policy tools included the use of supplemental budget allocations to provide extra spending on public health, social programmes for the vulnerable, tax relief for workers, households and businesses as well as loans and debt relief for companies (de Jong and Ho, 2020; Upadhaya *et al.*, 2020). However, the details and amounts of budget allocations to the health sector were often limited and therefore not readily comparable across countries.

For instance, by February 2021, the Government of Bangladesh had launched 23 stimulus packages with an estimated value of USD 14.6 billion [4.5% of estimated financial year (FY)20 GDP] (World Bank, 2021a). These primarily focused on alleviating the economic impacts of COVID-19, particularly on the garment sector, with only limited mention of the health sector beyond incentives for health workers (Raihan, 2020; World Bank, 2021a). However, others report that the Ministry of Health and Family Welfare (MOHFW) allocated USD 36.5 million for COVID-19 patient management, quarantine and isolation facilities, augmenting hospital capacity to provide COVID-19-related services (Akhter *et al.*, 2021).

To overcome these discrepancies, we referred to the IMF fiscal measures response database, which provides a consistent account of each country’s fiscal packages. Table 3 includes ‘above the line’ measures only, i.e. those that are reflected in the fiscal balance, government debt and increased borrowing needs in the short term (International Monetary Fund, 2020a). This includes additional spending (e.g. health services and unemployment benefits), capital grants and targeted transfers and tax measures provided through standard budget channels (International Monetary Fund, 2020a).

**Table 3.** IMF fiscal measures response database for selected SEAR countries, as of 17 March 2021

	Above the line measures: additional spending or foregone revenues by country since January 2020 to 17 March 2021					
	Subtotal		Health sector		Non-health sector	
Country	Billion	% GDP	Billion	% GDP	Billion	% GDP
Bangladesh	4.6	1.4	0.4	0.1	4.2	1.3
Bhutan	NA	NA	NA	NA	NA	NA
India	90	3.3	10	0.4	80	3.0
Indonesia	48	4.5	19	1.8	29	2.7
Maldives	0.3	6.9	0.1	2.6	0.2	4.3
Myanmar	0.9	1.1	0.2	0.2	0.7	0.8
Nepal	NA	NA	NA	NA	NA	NA
Sri Lanka	0.6	0.5	0.1	0.1	0.5	0.4
Thailand	41.2	8.2	NA	NA	NA	NA
Timor-Leste	0.3	15.8	NA	NA	NA	NA

Source: International Monetary Fund (2020a). Fiscal Monitor: Policies to Support People during the COVID-19 Pandemic. Washington: IMF.

#### Reallocation of existing resources

Several countries have freed up fiscal space for their pandemic response through repurposing non-urgent financing, reserves and emergency funds. In Indonesia, the Presidential Instruction (Inpres) No. 4 year 2020 was issued to reallocate the central budget towards the impending crisis and give local governments similar authority (Safitri *et al.*, 2021). More than 50% of the Family and Health Division budget was refocused for COVID-19 by cutting activities such as travel expenditures, meetings and honorariums and moving others onto virtual platforms (Soewondo *et al.*, 2021).

Similarly, in Thailand, the total government budget for FY 2020 (October 2019–September 2020) was revised with the unspent funds—particularly earmarked travel, meetings and other non-essential costs—recalled and reallocated towards COVID-19-related activities (Patcharanaruamol *et al.*, 2020). In Myanmar, up to 10% of the initial budget expenditure for the FY 2019–20 (excluding those implemented by foreign loans and grants) of each ministry was reallocated to the COVID-19 response, and unspent funds were returned to the General Reserve Fund (International Monetary Fund, 2020b). In total, 2631 billion Kyat (2.3% of GDP) was reallocated from line ministries to the General Reserve Fund (World Bank, 2021d).

Amongst Indian states, pay cuts for elected representatives and government officials were reportedly a popular move (Rao, 2020). The cuts exceeded 50% in many cases and were as high as 75% (for legislators in Telangana) (Rao, 2020). Some states also deferred a portion of their payroll to free up cash resources in the short term. Following the announcement by the Union government, many state governments announced a freeze of the ‘dearness allowance’ (an allowance paid to mitigate the effect of inflation) for government staff (Rao, 2020). However, information on how these reallocations affected health budgets was not available.

#### Revenue generation: mobilization of ‘new money’

SEAR countries implemented a range of policies to mobilize additional resources for the COVID-19 response, including selling assets and bonds, private donations to extrabudgetary funds and drawing on sovereign wealth funds. In India, the Central Government turned to the sale of natural resources and government assets as another revenue source (Jose *et al.*, 2020). In June 2020, the government announced an auction of 41 coal mines that were expected to attract capital investment of INR 330 million, reduce government expenditure on coal imports and increase state revenues by INR 200 million (Jose *et al.*, 2020).

Other countries sold bonds to generate resources for the COVID-19 response. In September 2020, The Royal Government of Bhutan announced its first sovereign bond to support the economy in recovery from the COVID-19 pandemic while diversifying financial sources (UNESCAP, 2020). The government completed the offering of a 3-year bond of Nu. 3000 million (or USD 41 million) at an annual coupon rate of 6.5% to support financing fiscal needs in responding to COVID-19 pandemic (UNESCAP, 2020). This was followed by a 10-year bond offering of USD 9.6 million in January 2021. In April 2020, Indonesia raised USD 4.3 billion for the COVID-19 response by selling a 50-year bond (the country’s largest ever) (Murdoch, 2020).

Four countries established extrabudgetary funds to support their pandemic response. Bhutan, Nepal and Sri Lanka established off-budget funds for donations from public, private and external sources (Rahim *et al.*, 2020). By July 2020, Sri Lanka’s off-budget fund had collected LKR 1.5 billion (USD 8.1 million) (ColomboPage, 2020a). India’s Prime Minister’s Citizen Assistance and Relief in Emergency Situations Fund had received over Rs 3000 crore in donations within 4 days after it was established on 27 March 2020 (Venkat, 2020).

#### Development partner funding

Multilateral donors announced significant resources to support efforts to contain and mitigate COVID-19 in SEAR countries, with Low- and middle-income countries (LMIC) in the region receiving considerably more support compared to countries such as Thailand, Maldives and Timor-Leste. Table 4 documents emergency funds to support national COVID-19 responses from the World Bank, Asian Development Bank (ADB) and IMF. However, very little information was available on how donor funding was used in country’s health responses, how quickly it was disbursed, whether it reached implementing organizations, intended populations and whether proposed activities were effectively implemented.

**Table 4.** External Financing from World Bank, Asian Development Bank and IMF, by country, publicly available by 1 March 2021 (USD)

Country	World Bank COVID-19 emergency response projects (USD)	ADB COVID-19 Active Response and Expenditure Support Programme (USD)	IMF rapid credit facility (USD)	IMF rapid financing instrument (USD)
Bangladesh	100 million	600 million	244 million	488 million
Bhutan	5 million	21 million	NA	NA
India	2 billion	1.5 billion	NA	NA
Indonesia	950 million	1.8 billion	NA	NA
Maldives	31.2 million	52.3 million	28.9 million	NA
Myanmar	50 million	250 million	86.1 million	172.3 million
Nepal	29 million	253 million	214 million	NA
Sri Lanka	128.6 million	3 million	NA	NA
Thailand	NA	1.5 billion	NA	NA
Timor-Leste	NA	1 million	NA	NA

Sources: World Bank Group’s Operational Response to COVID-19 (coronavirus)—Projects List (https://www.worldbank.org/en/about/what-we-do/brief/world-bank-group-operational-response-covid-19-coronavirus-projects-list); IMF COVID-19 Financial Assistance and Debt Service Relief (https://www.imf.org/en/Topics/imf-and-covid19/COVID-Lending-Tracker); ADB COVID-19 Active Response and Expenditure Support Programme (https://www.adb.org/what-we-do/covid19-coronavirus).

Notably, Timor-Leste’s response to COVID-19 has largely been ‘self-funded’, relying on a total of USD 333.2 million in extraordinary withdrawals from the Petroleum Fund (Jamal, 2021). By the end of FY 2020 (December) 76% of the allocation had been disbursed (Jamal, 2021). Others report that by August 2020, Timor-Leste had received the lowest level of external assistance among Pacific countries (Howes and Surandiran, 2020).

### Domain 2: policies to reduce financial barriers to seeking care

#### Subsidies for COVID-19 tests

In the early stages of the pandemic, all SEAR countries made COVID-19 tests available free of charge in the public sector. However, some countries first enforced restrictive eligibility criteria to ration available resources and rapidly scale-up testing capacity and personal protective equipment reserves. In Sri Lanka and Indonesia, for instance, tests were freely available at public facilities for cases with observable symptoms but not for asymptomatic cases or those without any contact history (Mathauer *et al.*, 2020; The Conversation, 2020). These restrictions were gradually loosened over time, and by March 2021, most countries had broad eligibility criteria for free COVID-19 tests in the public sector including migrants and, in some cases, foreigners.

Cost-sharing policies for COVID-19 tests changed rapidly over the course of the pandemic. In India, against a backdrop of mounting cases and limited testing capacity, the Indian Council of Medical Research initially recommended a cap of INR 4500 (USD 60) on co-payments for COVID-19 tests conducted in private facilities (Government of India, 2020). However, on 8 April, the Supreme Court ordered all tests to be carried out free without clarifying if and how private laboratories would be reimbursed (National Herald, 2020). Following a petition from the government to reconsider, on 13 April, the Supreme Court ruled that the government will reimburse private facilities for testing beneficiaries of the country’s flagship public health insurance scheme for lower socio-economic groups, AB-PMJAY (Ayushman Bharat Pradhan Mantri Jan Arogya Yojana) (The Business Standard, 2020b).

Only two countries opted to introduce co-payments for COVID-19 tests in the public sector. In June 2020, the Government of Bangladesh announced that all COVID-19 tests at government health facilities would incur a fee of BDT 200 (USD 2.4) and those conducted at a patient’s home would have a fee of 500 BDT (USD 5.9) (Kumar and Pinky). This policy was reportedly implemented to ‘avoid unnecessary tests’ (Cousins, 2020). These fees were subsequently reduced to USD 1.2 in August 2020 and remained in place as of 1 March 2021 (Tribune, 2020a). The introduction of the fee contributed to a reduction in the number of tests conducted per day from 18 426 (on 30 June 2020) to 10 759 (on 9 August 2020), equivalent to the second lowest ratio of number of tests to population size globally (The Business Standard, 2020a).

The numerous shifts in Nepal’s policy regarding COVID-19 testing demonstrate the challenge governments face in managing access to resources in a highly constrained fiscal setting. Initially, COVID-19 tests and treatment provided through public health facilities were free for all meeting the national testing guideline criteria (Government of Nepal, 2020). Yet, on 19 October 2020, the Federal Government announced a co-payment of Rs 2000 (USD 16.75) for COVID-19 tests in public hospitals with exemptions for the economically deprived, differently abled, single women, senior citizens and frontline health workers (Srivatsan, 2020). On 10 November 2020, the government reportedly removed the co-payment as case numbers rapidly increased; yet, reports from February 2021 suggest that a co-payment of Rs 1000 (USD 8.5) remains (Al Jazeera, 2020; Online Khabar, 2021).

#### Engagement of the private sector

The private sector played a significant role in the national response to COVID-19 in several countries. The ability of ministries of health to rapidly engage the private sector to provide COVID-related care influenced their ability to mount effective national responses to COVID-19. The pandemic clearly showed that countries with pre-existing mechanisms of private sector engagement were able to do this more efficiently than those without established mechanisms of engagement.

Thailand’s engagement of the private sector represents best practice in the region and was facilitated by a near-seamless integration of public and private care prior to the pandemic (World Health Organization, 2020d). Building on previous experience with SARS-CoV-1 and other infectious disease outbreaks, the Ministry of Public Health moved quickly to expand its cooperation and capacities across government ministries and the private sector (World Health Organization, 2020b). For instance, the Department of Disease Control produced guidelines and a protocol for case management that apply to both public and private hospitals. As part of this, private hospitals were required to report cases daily to the Centre for COVID-19 Situation Administration, chaired by the Prime Minister (O’Hanlon and Hellowel, 2020).

In other countries (Bangladesh, Nepal and Sri Lanka), the pandemic exposed limited public-private engagement and governance mechanisms. In these cases, engagement of the private sector appeared to have been hampered by a lack of trust as some governments initially refused to allow private hospitals to provide COVID-19-related care due to fears of profiteering. For example, on 18 March 2020, the Government of Bangladesh announced that private labs would not be allowed to perform COVID-19 tests due to concerns that with limited oversight they would use the opportunity to financially capitalize (Tajmim, 2020).

However, with a surge in demand and low testing rates, many governments were left with little option but to engage the private sector to expeditiously expand access by leveraging existing private testing and treatment facilities and resources. In Bangladesh, Sri Lanka and Nepal, a small number of private hospitals were allowed to provide COVID-19 testing subject to tight restrictions, and this number gradually expanded over time (WHO Country Office for Nepal, 2020; ColomboPage, 2020b). In Bangladesh, the number of approved private facilities gradually expanded from 3 to 34 facilities by 24 July 2020 (Hasan, 2020).

In India, reports suggest that the state’s ability to engage private providers was constrained by limited regulatory and purchasing capacity (Rao *et al.*, 2021; Shah, 2021). Despite this, the national flagship health insurance scheme AB-PMJAY vastly increased the number of hospitals empanelled under the scheme to provide free COVID-19 tests and treatment (Joseph *et al.*, 2021). The National Health Authority introduced Hospital Empanelment Module Lite, a new online system to rapidly onboard hospitals (Financial Express, 2020). Of the total facilities empanelled (*n* = 20 257) under the scheme in 2020, more than half (56%) were in the public sector, while 40% of facilities were private for profit, and 4% were private not-for-profit entities (Joseph *et al.*, 2021).

The introduction of price caps on private hospitals was a common policy response to these practices; yet, due to weak enforcement and regulatory capacities, there was limited evidence that these were effectual in reigning in adverse behaviour. In Sri Lanka, reports suggest that private hospitals commonly charged above the capped price of LKR 6000 (USD 32.5), up to 8800 (USD 47.9) (Charindra, 2020; Dissanayake, 2020). Similar accounts from Bangladesh, India, Indonesia and Nepal documented arbitrary pricing of COVID-19 tests and treatment by private facilities (The Conversation, 2020, Tribune, 2020b; IndiaSpend, 2020; The Kathmandu Post, 2020). The majority of these reports have emerged from India, leading some to suggest that the weak and fragmented regulatory landscape of Indian private health has made these behaviours more systemic (Williams *et al.*, 2021).

### Domain 3: changes in strategic purchasing arrangements

Responses by countries entailed a range of adjustments to purchasing arrangements. In April 2020, the Government of Indonesia passed Decree 238/2020, which established that the Ministry of Health (MOH) would be the main purchaser of COVID-related services, covering all hospitalized patients dating back to January 2020 (Pattnaik *et al.*, 2020a). The government opted to use a mixed purchasing system with the MOH responsible for developing the tariff and payment mechanism for COVID-19 patients and directly paying providers (Pattnaik *et al.*, 2020b). The National Social Insurance Agency (BPJS) assisted this process by receiving and verifying claims regarding COVID-19 from health facilities and submitting them to the MOH for payment (Pattnaik *et al.*, 2020b). All hospitals, regardless of whether they were contracted by the national health insurance scheme, submitted claims for COVID-19 through this newly established system. Reported challenges included uncontracted hospitals being unfamiliar with the claims system, delays in reimbursement and difficulties verifying claims due to the change in payment method (from a fixed amount per case to fee-for-service for COVID hospital care) (Pattnaik *et al.*, 2020a).

In Thailand, the cost of COVID-19 tests and treatment were covered by the Thai government’s three healthcare schemes: Universal Coverage Scheme [run by the National Health Security Office (NHSO)], Social Security Scheme (run by Social Security Office) and Civil Servant Medical Benefits (run by the Comptroller General’s Department) (Patcharanaruamol *et al.*, 2020). All Thais are reimbursed the cost of COVID-19 tests by the NHSO and for admission by their respective insurance scheme (Patcharanaruamol *et al.*, 2020). Hospitals received approximately 800 000 THB (USD 24 693) for providing treatment to a severe COVID-19 patient and around 400 000 THB (USD 12 346) for non-critical patients (National Health Security Office, 2021).

In Bangladesh, a contractual agreement was not established to facilitate private sector contracting, rather a simple agreement was reached between COVID-19 designated private facilities and the MOHFW to use fee-for-service as the reimbursement modality (Akhter *et al.*, 2021). Private facilities billed the Director General of Health Services under MOHFW, with itemized claims for patients managed at their facility. This arrangement required significant trust from both parties as there were no pre-negotiated rates and treatment protocols for high end care were not set (Akhter *et al.*, 2021).

In India, while many states involved the private sector in service provision for COVID-19, only some elected to pay for these services (Kabaniha *et al.*, 2021). For instance, in Odisha and West Bengal, no mechanisms existed prior to COVID-19 for the public sector to engage private providers, outside of AB-PMJAY. In Odisha, for the six private hospitals dedicated for treatment of COVID-19 (out of 26 hospitals in total), the state government used a mixed payment mechanism for hospital admission: per diem payments for each bed irrespective of whether it was used and monthly lump-sum payments to cover fixed costs (Government of Odisha, 2020). In West Bengal, where 55 of the 92 COVID-19 dedicated hospitals were private providers, fee-for-service payments were used to reimburse private facilities (Kabaniha *et al.*, 2021).

In Nepal, on 26 April 2020, the government made it mandatory for all public hospitals, medical colleges and selected private hospitals to establish COVID-19 clinics in designated areas of the hospitals (Ministry of Health and Population, 2021). A fee schedule for the private sector for COVID-related care was established through a costing exercise, which took into account the minimum quality standards. As per the directive, upon completion of COVID-19 treatment, any private, non-government, cooperative and community hospital could seek reimbursement from the government (Ministry of Health and Population, 2021).

#### Changes in public financial management systems

The unprecedented financial demands created by COVID-19 required rapid adaptation and reforms to PFM systems. Bhutan benefited from introducing PFM reforms prior to the pandemic. In 2019, the government introduced an online accounting and payment system to promote cashless and digital payments, which facilitated disbursement of the country’s income support programme (World Bank, 2021b). A number of reforms were subsequently introduced as part of the response to the pandemic including an Electronic Government Procurement System that supports government-wide procurement through an online platform (World Bank, 2021b).

In India, the Central Government introduced several PFM reforms to accelerate budget disbursement and flexibility, many of which were focused on supporting state governments. The MOHFW relaxed the virement of 10% to enable states to reallocate resources within the health system flexi-pool and allowed them to use untied funds for the development of isolation facilities (Kabaniha *et al.*, 2021). Most Indian states also relaxed their procurement laws and regulations to facilitate emergency spending, including dispensing partially or completely with elaborate tender processes; the waiving of supplier guarantee requirements and payment of 100% advances to suppliers (Rao, 2020). Most states attempted to constrain the impact of these measures by time-limiting them, capping their value or restricting them to certain categories of goods and services (Rao, 2020).

In Bangladesh, the government authorized facilities to reallocate unspent budget towards COVID-19-related activities and issued technical guidance to facilities on how to mount a COVID-19 response while maintaining routine service delivery (Akhter *et al.*, 2021). However, procedures to comply with the MOHFW ‘delegation of financial authority’ rules hindered rapid approval and utilization of funds (Akhter, 2021). Due to years of centralized control of financing functions, many health managers lacked the training and expertise to navigate the complex PFM requirements and unlock the funding available to their facility (Akhter *et al.*, 2021).

Similarly, in Indonesia, the lack of PFM capacity at the district level to revise budgets according to national government guidelines led to pools of funds for COVID-19 sitting at the national level (Soewondo *et al.*, 2021). Central ministries in charge of essential health services delayed issuing budget revision guidance to districts and frontline providers, leading to confusion on how to balance COVID-19 services with routine services (Akhter, 2021). The sheer number of funding streams, each with their own specific procedures and guidance, proved to be a major barrier for districts to quickly revise and submit budgets so that much-needed funds could be disbursed (Soewondo *et al.*, 2021).

Despite this, Indonesia increased budget execution by late 2020. As of July 2020, only 1.5% of the MOH budget had been disbursed due to the constraints in the verification process and bureaucratic delays, resulting in complaints from health workers (Ayuningtyas *et al.*, 2021). Yet, by the end of the year, the World Bank reported that 83% of the 2020 COVID-19 Fiscal Package had been spent, including 70.3% of the budget allocated to the health sector (World Bank, 2021c). Overall execution was reportedly improved by successive reallocation towards faster disbursing programmes.

## Discussion

This review provides a snapshot of the immediate health financing policies adopted by countries in the WHO SEAR in the first 12 months of the pandemic. While countries demonstrated a high degree of flexibility in responding to the COVID-19 pandemic, some were undermined by underlying systemic weaknesses, which should give countries an impetus for reform. The sheer number of emergency announcements to expand the funding and capacity of the health system demonstrate that the pandemic exposed pre-existing health financing policy weaknesses such as underinvestment and passive purchasing. Nevertheless, despite considerable contextual differences, some key lessons are evident from SEAR countries’ management of the financing challenges created by the COVID-19 pandemic.

First, governments were forced to look at all possible sources of financing to fund their crisis response. This included budget reallocations, borrowing from multilateral organizations, reserve funds and innovative means such as the issuance of government bonds (Bhutan and Indonesia), expansion of public sector funding limits spending limits (India) and community and private sector donations to extrabudgetary funds (Bhutan, India, Nepal and Sri Lanka). However, it is unlikely that any of these strategies represent sustainable solutions for expanding the funding envelope for health. Indeed, one concern, particularly for options that entail greater public sector debt and extending spending limits, is that these one-off measures may, over coming years, create debt burdens that hamper the ability of countries to promote economic development and health system capacity. Similar concern can be expressed that some of the substantial and seemingly arbitrary cuts in existing budgets may have disrupted core public health activities, with future health and economic consequences. Funding pandemic preparedness as a global public good with rapid access to emergency financing for low- and middle-income countries may hold promise for countries to avoid these challenges in the future (Sirleaf and Clark, 2021).

While access to free testing for COVID-19 was universally adopted in most countries, in some cases (Bangladesh and Nepal), due to financial pressures, policies were enacted that allowed for the introduction of user fees. Furthermore, across a number of settings, there was predictably evidence of informal fees being charged at facilities where testing was nominally free. These fees—whether formal or informal—potentially created barriers to testing that enable disease transmission to occur unmonitored and unchecked. As acknowledged by the turnaround in policy in Bangladesh, user fees for testing can be counterproductive. During an outbreak, the public good aspect of such testing is critical and substantially outweighs the value of cost-recovery to government. In such circumstances, there seems to be an overwhelming case for such services—and perhaps more broadly other areas of health spending with significant public good (e.g. other vaccinations, infectious disease testing and treatment)—to be incorporated into benefit packages and be provided universally free of charge (Soucat and Kickbusch, 2020).

Health systems that have had well-developed institutions for strategic purchasing (including engaging with the private sector) were shown to be able to respond more rapidly and effectively in harnessing this capacity to support the COVID-19 response. This was the case in Thailand, which seemed to have benefited from previous experience with the SARS-CoV-1 outbreak and an efficient claims management and reimbursement agency. In other settings, governments were challenged to establish and manage effective purchasing arrangements during the crisis with limited experience and capacity. Limited regulatory and enforcement capacity was also evident as nearly all countries were challenged to reign in opportunistic behaviour in the private sector through the introduction of price caps on COVID-related services. As others suggest, these experiences highlight the need to reform and strengthen regulatory systems and, in doing so, rethink the role of the private health sector in the pursuit of universal health coverage (Williams *et al.*, 2021).

Lastly, the unprecedented financial demands created by COVID-19 spurred countries in the region to introduce rapid reforms to PFM systems and relax rules around the procurement of services. Inevitably, these changes also raise legitimate concerns about accountability and fiscal transparency; yet, they also have the potential to create an enduring legacy, help health systems to transition out of the pandemic and possibly prepare for the next crisis. Going forward, countries will need to monitor the impacts of these PFM reforms and use them as lessons to develop plans and standard operating procedures ahead of time that promote flexibility, while creating or maintaining checks and balances.

Limitations of this review should be noted. First, in the context of limited information and rapidly evolving policy responses, it was necessary for the review to include sources more diverse than peer-reviewed journal articles, such as grey literature, government reports and news media. While these sources were carefully appraised, this search strategy may pose difficulties for replication. Second, many of the reported policies reflect public announcements rather than implemented reforms and disbursements. Due to the timing of this review, it was not possible to measure actual disbursements or assess the impact of the health financing policies. An important task going forward is to ensure that these financing responses are rigorously monitored and evaluated.

## Conclusion

In the first 12 months of the pandemic (March 2020–21), countries in the SEAR demonstrated flexibility by announcing a range of health financing policy responses. This review highlights the various means employed for revenue raising, the importance of government stewardship of multiple actors to ensure the availability of quality and affordable services as well as the rapid adaptation of purchasing arrangements. Nevertheless, the pandemic exposed pre-existing health financing policy weaknesses such as underinvestment, inadequate regulatory capacity and passive purchasing, which should give countries an impetus for reform towards more resilient health systems. Ongoing evaluation and monitoring will be needed to understand the long-term implications of such policy changes and how they protect progress towards the objectives of universal health coverage.

## Data Availability

The data underlying this article will be shared on reasonable request to the corresponding author.

## List of abbreviations

AB-PMJAY: Ayushman Bharat Pradhan Mantri Jan Arogya Yojana

BDT: Bangladeshi Taka

COVID-19, SARS-CoV-2: Coronavirus Disease 2019

INR: Indian rupee

LKR: Sri Lankan Rupee

Nu: Bhutanese ngultrum

PFM: Public financial management

SEAR: South-East Asia Region

THB: Thai Baht

USD: US Dollar

WHO: World Health Organization

## Reflexivity statement

The author group is gender-balanced (four females and four males) and spans multiple levels of seniority from students, early career researchers, a professor and professional staff including a Regional Adviser for Health Financing and Governance. The group represents a collaboration between The George Institute for Global Health and the WHO South-East Asia Regional Office, which aimed to produce evidence that supports policy and practice in the region. Authors from The George Institute possess extensive experience conducting evidence reviews, while those from WHO provide specialized in-depth experience and contextual knowledge of health financing in the South-East Asia Region. Some authors were based in Australia and therefore separate from the immediate context, whereas others were located in country offices and could support contextualization and representativeness of findings.
